# Non-invasive digestion monitoring with an FDA-cleared wearable biosensor: further validation for use in tracking food ingestion

**DOI:** 10.1093/gastro/goaa097

**Published:** 2021-01-30

**Authors:** Erica R Cohen, Mayra Lopez, Brennan M R Spiegel, Christopher V Almario

**Affiliations:** 1 Karsh Division of Gastroenterology and Hepatology, Cedars-Sinai Medical Center, Los Angeles, CA, USA; 2 Cedars-Sinai Center for Outcomes Research and Education (CS-CORE), Los Angeles, CA, USA; 3 Division of Health Services Research, Cedars-Sinai Medical Center, Los Angeles, CA, USA; 4 Department of Health Policy and Management, UCLA Fielding School of Public Health, Los Angeles, CA, USA; 5 Division of Informatics, Cedars-Sinai Medical Center, Los Angeles, CA, USA

## Introduction

Obesity affects one-third of the US population and accounts for $200 billion/year in expenditures [1]. Diet and exercise form the cornerstone of management, yet are often ineffective [2]. Mobile health applications (mHealth apps) can augment traditional strategies; Gill and Panda [3] demonstrated that obese patients can modify their eating behavior and lose weight using an mHealth app to track food intake. While promising, these apps typically require active data input and currently lack passively collected, physiologic input to support sustained behavior change.

Acoustic gastrointestinal surveillance (AGIS) using a wearable biosensor (AbStats; GI Logic, Pasadena, CA) is cleared by the US Food and Drug Administration (FDA) as a validated technique to non-invasively measure intestinal motility [4–6]. Two biosensors adhere to the external abdominal wall and analyse intestinal sounds (Supplementary Figure 1), which are counted and time-averaged per minute to yield an “intestinal rate” (IR). By listening to bowel contractions and counting them using an app, AGIS enables patients to monitor digestion continuously using a wearable “gut speedometer.” In prior studies, AGIS distinguished post-operative ileus from non-ileus surgical patients and predicted ileus onset with 97% accuracy [5, 6]. We now seek to determine whether AGIS biosensors can distinguish small, calorically light meals from large, calorically dense meals in healthy subjects in a proof-of-concept study.

## Patients and methods

**Figure 1.**
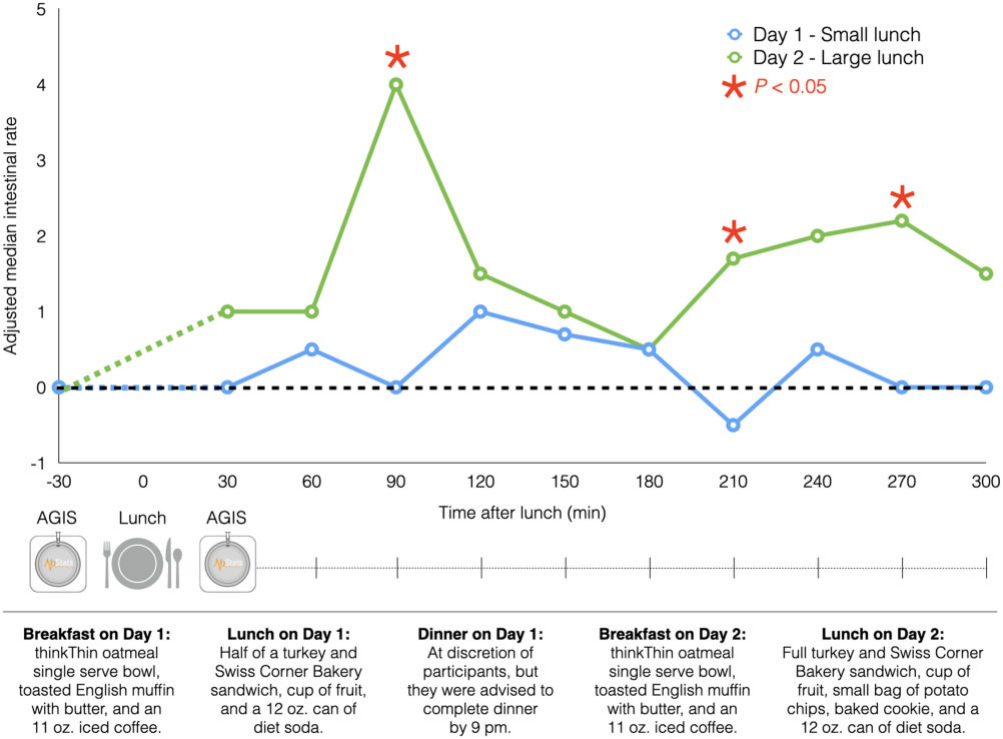
Adjusted median intestinal rates after small and large standardized meals. At 90, 210, and 270 min post meal, the intestinal rates are significantly higher after a large meal than those measured after a small meal.

We recruited 25 healthy volunteers aged 18–65 years between February 2017 and July 2017. Subjects with conditions that affect gastrointestinal motility, prior gastrointestinal surgery, or who were taking medications affecting motility were excluded. Each subject who provided informed consent received four standardized meals to consume over a 2-day period (Figure 1). The same 466-kilocalorie breakfast was consumed on both days. Lunch was eaten 4–5 hours afterwards and varied between Day 1 (small 360-kilocalorie meal) and Day 2 (large 1,110-kilocalorie meal). Dinner was at the participants’ discretion and they were instructed to complete it by 9 pm.

Subjects recorded their IR using the biosensors 30 min before lunch and then every 30 min after for 5 h using an app (Figure 1). Pre-prandial-fasting IR was normalized at zero and post-prandial rates were scaled accordingly. We used the Wilcoxon signed-rank test to compare post-prandial IR between small vs large meals at each time point. We further compared cumulative IR between post-prandial periods that represented overall digestive effort. This study was by the Cedars-Sinai Institutional Review Board (Pro45465).

## Results

Twenty-five participants completed the study; the median age was 27 years (range, 21–56 years) and 60% were male. We noted the following racial/ethnic breakdown: 36% non-Hispanic White, 24% Latinx, 8% non-Hispanic Black, and 32% other/multiracial. The median body mass index was 24.2 kg/m^2^ (range, 19.5–34.5 kg/m^2^). Two subjects had co-morbidities (deafness and folliculitis) and one was taking clindamycin.

Figure 1 presents the IR measurements after the small vs large meals. Median measurements after the large meal statistically diverged from those after the small meal at 90 min (4.0/min [range, −3.1 to 18.9/min] vs 0.0/min [range, −7.4 to 11.0/-min], change from baseline, *P* = 0.021), 210 min (1.7/min [range, −8.3 to 16.3/min] vs –0.5/min [range, −10.4 to 10.1/min], *P* = 0.026), and 270 min (2.2/min [range, −2.0 to 20.0/min] vs 0.0/min [range, −10.4 to 7.2/min], *P* = 0.018) post meal. There was a higher median cumulative IR after the large (615.0 [range, −829.5 to 3,490.5]) vs small (195 [range, −1,846.5 to 2,006.3]) lunches (*P* = 0.048).

## Discussion

Using a non-invasive, FDA-cleared wearable biosensor, we collected normative data to establish the effect of varying caloric intake on intestinal activity in healthy subjects. We found that a computer-aided listening device can distinguish between small and large meals in as early as 90 min. High-calorie meals generate higher, sustained intestinal activity than low-calorie meals, as measured and presented in an app.

Patients with obesity generally consume too many calories, too quickly, and too frequently [2]. Obese individuals often are not aware that they are still digesting a previous meal when initiating another meal. Feedback from wearable biosensors may be useful to guide the optimal timing of meals. For example, if a meal ingested earlier in the day were still undergoing active digestion, then it would be premature to ingest another meal. Here, a wearable digestion monitor might guide users by placing them in “red,” “yellow,” and “green” eating zones, based on dynamic changes in digestion.

While this was a proof-of-concept study, there are limitations. First, we had a limited number of participants and were unable to assess for racial/ethnic differences in the impact of caloric intake on AGIS-measured IR. We were also underpowered to detect differences between normal-weight and overweight/obese individuals; abdominal fat may impact AGIS IR recordings. Future studies addressing these issues are needed. Second, we could not determine the source of the intra-abdominal sounds (i.e. stomach vs small intestine vs colon) using AGIS. Of note, two IR peaks were noted at 90 and 270 min after consuming the large meal; the first peak may reflect gastric/upper-small-intestinal contractions while the second peak may represent distal-small-bowel/colonic contractions or a phase II/III migrating motor complex. Studies correlating AGIS IR data to concurrent radionuclide transit data are needed to definitively assess localization. Third, we only compared IRs between large (1,110-kilocalorie) and small (360-kilocalorie) meals. Future research should examine the effect of intermediate-sized meals, meals of varying volume and composition, and beverages on IRs. Fourth, we only examined IRs after a standardized lunch consumed after a standardized breakfast. Subsequent studies should assess the impact of other meal timings on the IR. Finally, follow-up studies should examine whether migrating motor complexes occurring close to meal initiation impact post-meal IRs; this will be feasible once a wireless AGIS is developed, allowing continuous IR monitoring throughout the day.

These results lay the groundwork for understanding how a wearable biosensor might enable physiologically based ingestion guidance to complement traditional obesity management. AGIS may also prove useful for augmenting the usual care for gastrointestinal diseases, such as irritable bowel syndrome, Crohn’s disease, and ulcerative colitis, among others. Future research will evaluate intestinal activity over longer periods, evaluate a wider range of meal types, and test the impact of biosensor-tailored meal intervals on obesity outcomes.

## Supplementary Data

Supplementary data is available at *Gastroenterology Report* online.

## Authors’ contributions

E.C., M.L., B.M.R.S., and C.V.A. made substantial contributions to the conception and design of the work; acquisition, analysis, and interpretation of data for the work; and drafting of the work and revising it critically for important intellectual content. All authors read and approved the final manuscript.

## Supplementary Material

goaa097_Supplementary_Figure_1
