# Supplementary material for: Non-invasive digestion monitoring with an FDA-cleared wearable biosensor: further validation for use in tracking food ingestion
Source: Gastroenterol Rep (Oxf). 2021 Jan 30;9(5):475–7. doi: 10.1093/gastro/goaa097 (PMC8560032; doi:10.1093/gastro/goaa097)

**SUPPLEMENTARY FIGURE 1**. AGIS system. (A) AGIS sensor with Tegaderm bandage; (B) sensor with the adhesive exposed and is ready for application; (C) AGIS disposable sensors applied to abdominal wall of a participant; (D) bedside computer depicting AGIS-derived intestinal rate, defined as intestinal acoustic events per minute. The AGIS sensor supports a microphone array. The sensor moves readily with the subject’s breathing and movement. Each sensor is slightly larger than a U.S. quarter in size. The embedded microphone is miniaturized, low profile, yet highly accurate and capable of effective noise cancelation of false signals (e.g., external sound or rubbing on skin or clothes).


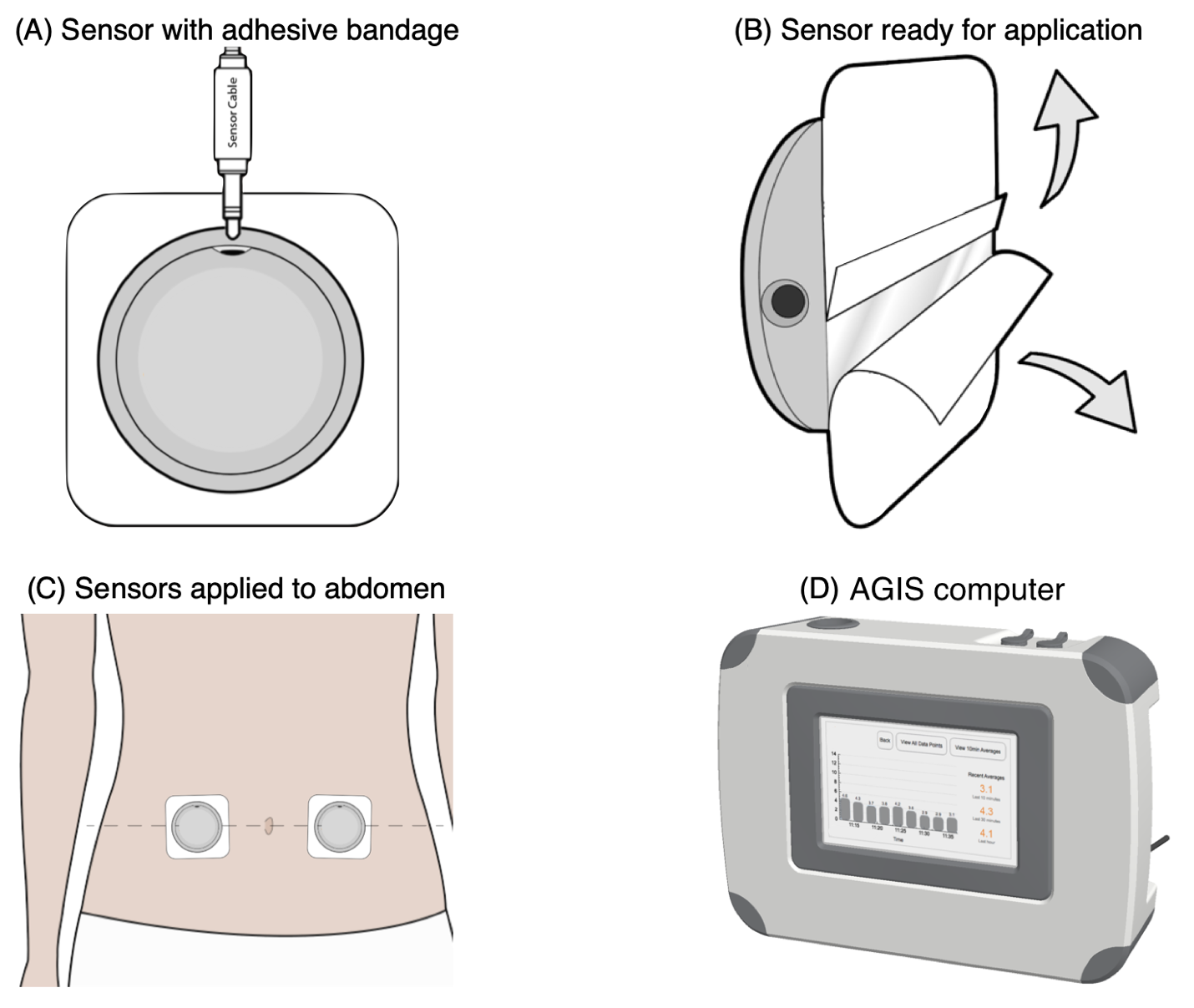

Supplement: goaa097_Supplementary_Figure_1 [file goaa097_supplementary_figure_1.docx]
